# Invalid Children's Aid Association

**Published:** 1889-11-16

**Authors:** 


### Invalid Children's Aid Association.

Some critics are angry with the present generation for knowing and saying that it has advanced upon the standard of its predecessors in the matter of benevolent work amongst the poor. Well, boasting is not a good thing, but false modesty is not much better. Let anyone with a heart read about the work of the Invalid Children's Aid Association, and how can he resist a feeling of satisfaction and delight that an effort is made to give a real friend to every crippled child amongst the London poor? We do not congratulate ourselves merely that more is done for the poor, but especially upon the spirit in which it is done. Mechanical laws (and mechanical officers) of relief are infinitely better than none, but the personal service—voluntary, kindly, unwearying—of those in a position to offer it is the best of all. The Hon. Dudley Fortescue says the ambition of this association is "that the day should come when not one suffering child in London should be without his friend, whose object it would be to ameliorate his condition and brighten his existence." This does not overstate the fact, and what is being done at this moment for over 500 invalid and crippled childrcn is the best commentary upon what it is a declared intention to accomplish.

The Invalid Children's Aid Association is not so young as one might suppose. The work was carried on for a time under the direct supervision of the Charity Organisation Society, but was very properly considered worthy of an independent existence, and the development to be looked for through a separate establishment of its own. Its headquarters are at 18, Buckingham-street, Strand, and its officers there (almost exclusively volunteers) have found its work trebled in less than a year. There is a great future before it.

We give an official description of the nature of the actual help now rendered: "If a child ought to be accompanied to a hospital by a visitor competent to understand the doctor's instructions, and to see them carried out at home; if a surgical appliance is to be provided, the purpose and proper management of which would be readily apprehended by a person of ordinary intelligence and education, whilst, on the other hand, its usefulness would be seriously lessened, if not altogether destroyed, by the stupidity and unhandiness of a very ignorant parent; if on being discharged from a hospital the patient should, in addition to the non-professional visitor, require the daily or occasional visits of a trained nurse; if a spinal carriage or wheel chair should be lent; if country or seaside change be needed to complete the cure; if admission, by payment, into a special hospital or home for incurables be desired; if medical comforts or appliances should be supplied in the child's own home; if the managers of a convalescent home or hospital in the country are sending back to London a patient who still needs treatment, nursing care, or befriending generally; if a minister, district visitor, Poor Law or School Board official, or anyone in contact with the poor, comes across a sick or crippled child whose case seems at least to need considering, that it may be referred to the proper agency or otherwise suitably dealt with—these and similar contingencies we seek to provide for, so far as circumstances and our funds will allow."

Our readers will appreciate the extent and usefulness of a plan like this, and the list of "work done" shows what a boon is conferred upon parents, and, above all, on the suffering children, by this admirable society. Most people will understand the significance of notes like these: "Was 'convalesced' by his visitor, and has recovered"; "Kept by the association in the country for six months"; "The carriage enables him to be in the open air, listening to the voices of the children, his great delight. Also to be taken by his mother on her errands."

Three special things may be named which press hard on the children of the London poor, and bring so many of them within the scope of this Society's beneficence: (1) Predisposition to disease from unhealthy antecedents and surroundings, from insufficient food, and ignorant management. (2) Special liability to accidents from insufficient "minding" and treacherous "stairs"—hence, spinal disease and its consequent humpback, hip disease, and its crippling deformities. (3) Negligent or ignorant "convalescing" in their own homes, sometimes with the result that an operation which, under favourable circumstances, might lead to recovery, issues, perhaps, in the child being crippled and maimed for life. Here there is surely need for some benevolent organisation acting on a large scale, and according to the methods which kindness, patience, gentleness, and charity will suggest. Great stress is laid by the society upon constant watching of cases, till all the benefits have been gained which may be expected from operations and other hospital treatment. As Dr. Octavius Sturges has put it: "Hospitals, designed for acute cases, could not, owing to the demand for beds, keep within their walls patients who needed only rest, good nursing, and careful diet. Many, therefore, of their little patients had to be discharged who still needed the most prudent and assiduous attention, together with appliances and comforts of different sorts, unattainable, for the most part, in a 'poor man's home." Seeing that this association provides precisely the supervision essential to the child's recovery, one need not wonder that Dr. Sturges pronounces it to be exactly what is needed to strengthen, widen, and altogether improve the work of children's hospitals.

The Society desires to supplement—not to supplant—existing agencies; and, whilst willing to be useful as a centre of information, and to serve also as a clearing-house at which invalid children may be assigned to the societies designed to deal with them, and to find for any afflicted child that which, perhaps, is the chief boon of all, *a friend* —still reminds those likely to appeal to them that adequate means at hand are not to be overlooked; it is not to be forgotten that hospitals, for example, have, for the most part, Samaritan Funds, some have convalescent homes of their own, and arrangements for providing surgical appliances. These agencies utilised, there will still be abundant work for a society aiming at the supervision and assistance of the invalid and crippled children of the London poor.

Our one purpose in this article is to help in making this Society better known, for we feel, with Lord Aberdeen, that its work needs only to be understood to secure it thorough support. We have not written without full investigation of what is being done, and hope we may at least have brought out some features of the work which will appeal to all wishing to help the poor in a systematic and really advantageous way. "The value," we hear on competent authority, "of the assistance given to the little ones is incalculable. It is systematic—not mere spasmodic individual efforts. It is co-operative, bringing different resources to bear upon each case as required. It draws out personal tenderness and sympathy, each visitor having one or two special cases to watch, help, and contrive for, and it is under careful *surveillance*—every case being periodically reported on and enquired into." We may add that everything possible is done to strengthen the hands of medical men and others engaged in hospital work. Similar co-operation is offered with all engaged in charitable work amongst the poor. But, perhaps, the great achievement of the Society is the disinterested and self-denying labours of its army of voluntary visitors. Here is a typical case:

"A semi-imbecile girl, who was found by the visitor, with her mother and two other children, in a deplorably destitute condition; half-clad, half-starved, in an underground kitchen, where a bed, a cot, and a chair represented the furniture, with absolutely no food and no prospect of getting any, every available article having been pawned. The history of the family was very sad. The father had died insane; one child had been burnt whilst the mother was away at work; her eysight was now failing, in part because of the conditions under which she lived; and yet she could not leave the house to earn anything, someone being always wanted to watch the afflicted child. The woman's character was good, and, thanks to the energy of the visitor, she and her children have been raised from the almost hopeless condition in which they were found. One boy is in a training home, the girl is boarded out with her other brother, and the mother is in a very respectable situation. The necessary funds have been provided, partly by friends of the visitor, partly by the Charity Organisation Society, and partly by this association."

Columns might be filled with similarly-gratifying records, but the best course will be to write to the honorary secretary, 18, Buckingham-street, Strand, W.C., for details; he will gladly supply them. It will be all the better if one can offer help in any of the many forms in which it will be welcomed. The spinal carriages and surgical appliances so largely used are costly, but once supplied, the liability is at an end. The prolonged periods of "convalescing"—involving residence in a pay hospital or home, or boarding out in the country or by the sea—also represents a very considerable expenditure. Many who cannot act as visitors might be ready to bear the expense of some one urgent case in this stage of slow recovery, and a "golden book" is kept for the names of those willing to give this kind of assistance. More visitors are also required, and ladies who have had some training as nurses are reminded that even if unable to visit habitually, their reports on occasional bad cases would be of special value. It may not be amiss to add that the Society is glad to receive "letters" for hospitals and convalescent homes, old spinal carriages, wheel chairs, perambulators, air and water cushions, children's clothes, cots, and mattresses.

